# Breast Course for Nurses: Educating Health Care Workers to Perform Clinical Breast Screening

**DOI:** 10.1200/JGO.2016.008128

**Published:** 2017-04-27

**Authors:** Jenny Edge, Lieske Wegelin, Dave Woods

**Affiliations:** **Jenny Edge** and **Lieske Wegelin**, Christiaan Barnard Memorial Hospital; and **Dave Woods**, Red Cross Children’s Hospital, Cape Town, South Africa

## TO THE EDITOR:

In many places in Africa, the first contact with a health professional for women with breast complaints is a nurse in a primary clinic. Over the past decades, there has been little formal breast training for nurses because the emphasis has been on the prevention and management of infectious diseases. However, as the incidence of infectious diseases falls, the WHO predicts that mortality due to noncommunicable diseases in the developing world will increase by 17% in the next 10 years. This will have a major impact on an already overburdened health system.^1^ Similarly, Goss et al^2^ have predicted that the prevalence of cancer in developing countries could increase by 90% by 2030. In many high-income countries, mammographic screening programs are offered. However, some countries, such as Switzerland, are reviewing their programs.^3^

Mammographic screening programs are not appropriate or feasible for the majority of low-income countries. In many African countries, there is minimal access to mammography equipment, and it is expensive. Although there are few accurate statistics for South Africa, an estimated 54% of women with breast cancer present with locally advanced disease. In a study from Baragwanath Hospital in Johannesburg, South Africa, it was found that women who lived further away from the breast clinic were diagnosed at a later stage.^4^

Health care services in South Africa are a mixture of those seen in low- and high-income countries. A different model of breast screening is necessary to improve the general well-being of the majority of women who rely on state health services.

Clinical breast examination has been studied as a modality of screening for cancer with variable results. In Canada, where clinical breast examination was compared with mammographic examination, the mortality from breast cancer after 20 years was similar in both groups, although more cancers were diagnosed in the mammographically screened population.^5^ In Sudan, nurses and volunteers were trained to perform clinical breast examinations on all women > 18 years of age in a screened population. The incidence and stage of breast cancer were compared with that in an unscreened population. Results showed that clinical screening resulted in women being diagnosed with earlier-stage breast cancer.^6^

To address the need for competent nurses in South Africa who are able to provide clinical breast screening, the Breast Course for Nurses (BCN) was developed. The course content of the BCN is based on the *Breast Care* course book, which is part of the Bettercare series.^7^ The Bettercare series of course books is written for nurses working in primary clinics and is designed to promote self-learning. The topics covered in each book are divided into small, easy-to-understand sections. The language used is tailored to nurses whose first language is not English. The *Breast Cancer* course book is divided into eight chapters. Three chapters are devoted to assessment of the breast and changes found in the normal breast, three chapters cover the principles of breast cancer care, one chapter covers investigations, and one chapter covers palliative care. The book was written in collaboration with professionals in breast cancer management in South Africa.

The BCN combines a distance education component using the *Breast Care* course book with a residential course. The residential course places the theory learned from the course book into practice. It is divided into three modules. Each module is progressively more specialized. Lay volunteers, community care workers, and registered nurses complete module 1, and doctors and the oncology sisters complete module 2. (Module 3 covers the material in module 1 in an abbreviated form.) The aim is to enable health care workers to differentiate between women who need to be referred for additional evaluation and women who can be safely managed locally.

One of the aims of the course is to make care for women with breast problems more accessible. The course teaches clear guidelines about when a woman with a breast lump needs to be referred. For example, the recommendation is that any women ≤ 25 years of age with no significant family history and with a new breast mass < 5 cm can be followed up clinically in 4 months, reducing the burden on the breast clinic. Should the mass increase in size, she should be referred for investigation. However, for any woman > 25 years of age with a new mass, investigation is mandatory.

The WHO conducted a survey on the availability of palliative care globally.^8^ They found no evidence of any form of palliative care in 22 African countries. Each course invites local health care providers to teach the principles of palliative care with the four drugs most commonly available (acetaminophen, morphine, amitriptyline, and ibuprofen).

Lymphedema is poorly managed in much of southern Africa, with few facilities available. One of the founders of the BCN is a trained lymphedema therapist. Lymphedema is not recognized as a disease in most African countries and is thought to be a normal occurrence for women with advanced breast cancer or after breast cancer surgery. Although South Africa is fortunate to have over 70 registered lymphedema therapists, Zimbabwe currently has only two, and there is no documented evidence of any lymphedema therapists in any other African countries.^9^

Lymphedema management can be expensive. In urban South Africa, patients with lymphedema may be referred to a therapist outside the cities, because few facilities exist. The BCN aims to educate nurses about lymphedema, how to recognize it, and how to manage and prevent it through simple exercises and breathing techniques.

The emphasis of the BCN course is on learning rather than teaching. Most of the faculty in the residential course are local health care providers. Therefore, although the core content of the course remains the same wherever the course is taught, the information becomes applicable for the available resources. It can also be adapted, depending on the type of health care workers being trained.

Since 2012, the BCN has been taught in South Africa on numerous occasions (Cape Town, Johannesburg, Durban, and Port Elizabeth), as well as in Malawi (Lilongwe), Zimbabwe (Harare and Bulawayo), and Namibia (Windhoek and Ongwediva). To date, over 600 health care providers have successfully completed the course.

One of the advantages of the BCN in its current format is its flexibility, allowing adaptation to local conditions. All participants complete a multiple-choice test, which is based on the *Breast Care* course book, before attending the residential course. At the moment, all participants receive a certificate of completion if they complete the multiple-choice test and attend the course. We are continuing to discuss the possibility of an end-of-course examination. Application has been made to the OCSA Academy of Excellence for registration for the BCN.

The learning methodology used in the *Breast Care* course book is the same methodology widely used and extensively evaluated in the Perinatal Education Program over the past two decades in South Africa.^10,11^ Nurses fill in an assessment form at the beginning of the course and are asked to complete the assessment again 6 months later. We are gradually accumulating data from participants. A study is being performed to determine whether women with breast cancer are being diagnosed at an earlier stage.

The BCN has many challenges but has been developed by health care practitioners in South Africa for the training of health care workers throughout southern Africa. The course is offered to health departments on request. Because local faculty are actively involved, there has not been much resistance to the content offered.

The stage of diagnosis of women with breast cancer is dependent on many factors. It is hoped that by educating nurses in primary clinics, women with breast cancer will be referred more appropriately and in a more timely manner.
